# *Anopheles stephensi* larval habitat superproductivity and its relevance for larval source management in Ethiopia

**DOI:** 10.1186/s12936-025-05589-y

**Published:** 2025-10-22

**Authors:** Solomon Yared, Dereje Dengela, Peter Mumba, Sheleme Chibsa, Sarah Zohdy, Seth R. Irish, Melissa Yoshimizu, Meshesha Balkew, Albert Akuno, T. Alex Perkins, Gonzalo M. Vazquez-Prokopec

**Affiliations:** 1 Department of Biology, Jigjiga University, Jigjiga, Ethiopia; 2 Abt Global, PMI Evolve Project, Addis Ababa, Ethiopia; 3 U.S. President’s Malaria Initiative, USAID, Addis Ababa, Ethiopia; 4 Entomology Branch, U.S. President’s Malaria Initiative, U.S. Centers for Disease Control and Prevention, Atlanta, GA USA; 5 U.S. President’s Malaria Initiative, USAID, Washington, DC, USA; 6 Department of Biological Sciences, University of Notre Dame, Notre Dame, IN 46556 USA; 7 Department of Environmental Sciences, Emory University, Atlanta, GA 30322 USA

**Keywords:** Population dynamics, Rainfall, Larvicide, Heterogeneity

## Abstract

**Background:**

The invasion of Africa by *Anopheles stephensi* poses a significant threat to malaria elimination. Unlike African Anopheline species, *An. stephensi* may be less impacted by rainfall seasonality due to its predominance within artificial containers. To test this assumption, the seasonal transition of an established population from eastern Ethiopia between rainy and dry periods was quantified. Secondary objectives included the theoretical quantification of the impact of habitat seasonality on the efficacy of larval control, and the study of habitat overlap between *An. stephensi* and *Aedes aegypti*.

**Methods:**

The study was conducted in the city of Kebri Dehar, Somali Region of Ethiopia. Standard dipping was conducted in 100 houses during the peak rainy season (November 2020). All habitats with water at that survey were followed monthly until the peak of the dry season (February 2021). Positivity and productivity of *An. stephensi* and *Ae. aegypti* were quantified. Negative binomial and Pareto fraction analyses characterized heterogeneity in productivity by habitat type. A two-patch metapopulation model quantified the impact of rainfall seasonality and heterogeneity in larval habitat productivity on the entomological impact of a larval source management campaign.

**Results:**

As the dry season progressed, *An. stephensi* productivity significantly concentrated in large water reservoirs (for drinking and construction). Larval productivity was significantly overdispersed (parameter k of negative binomial distribution ranging between 0.28 and 0.43). Pareto distribution analyses estimated that 77% of all larvae originated from 23% of the sites. Such superproductive sites were primarily water cisterns used for residential or construction purposes. The most productive *An. stephensi* habitats were not so for *Ae. aegypti*, which infested primarily tires and small containers. The metapopulation model predicted that larval control targeted on superproductive water reservoirs, when implemented at coverages higher than 60%, may lead to *An. stephensi* elimination.

**Conclusions:**

This study highlights the role of environmental variability in regulating *An. stephensi* populations and opens the window for the deployment of control strategies that exploit major mosquito population bottlenecks. The partial overlap between *An. stephensi* and *Ae. aegypti*, particularly during the dry season, point to challenges to control both species if only large containers are treated.

**Supplementary Information:**

The online version contains supplementary material available at 10.1186/s12936-025-05589-y.

## Background

As the mosquito *Anopheles stephensi* continues its invasive range expansion within northern Africa [1], major questions about its ecology, bionomics, and epidemiological role still remain [2]. Current distribution in Africa includes Djibouti [3], Ethiopia [4], Ghana [5], Kenya [6], Sudan [7], Somaliland [8], and Nigeria. In its native range (centred in India, Iran, Pakistan, and the Arabian Peninsula) *An. stephensi* is a major malaria vector found to be highly competent to both *Plasmodium falciparum* and *Plasmodium vivax* [9]. It is estimated that 12–15% of all urban malaria cases in India are vectored by *An. stephensi* [10]. In Africa, the rapid increase in malaria case reports in Djibouti between 2017 and 2020, five years after the first detection of *An. stephensi,* [11], sent an alarming message about the potential for increased malaria incidence in the context of major and sustained reductions in morbidity and mortality throughout the continent [12–14]. Observational studies suggest increased malaria rates are beginning to occur within Ethiopia, however, there seems to be heterogeneity in urban malaria increases in sites where *An. stephensi* has been detected [9, 15–17]. A challenge with assessing the epidemiological role of *An. stephensi* is often due to the limited knowledge of human-mosquito contacts, which depend on the level of productivity of larval habitats near human habitations and the flight and resting behaviour of mosquitoes.

In its native habitat, *An. stephensi* exists as three biological forms that seem to be separated by environmental factors (the ‘type’ form is highly adapted to urban centres and feeds on humans, the ‘intermediate’ form is found in peri-urban settings, and the ‘mysorensis’ form inhabits rural habitats and tends to feed more frequently on animals [18]). Reports from Africa suggest that the species is primarily urban, although non-urban detections have also been seen [19]. In fact, urbanization itself appears to be favoring *An. stephensi* establishment via the availability of large and permanent habitats in construction sites due to water storage associated with brick curing [20]. Interestingly, reports of larval habitats for *An. stephensi* in Ethiopia have either focused on cross-sectional characterizations during the dry season [20] or rainy season [4, 7, 21–23]. Unlike native *Anopheles* vectors, the ability for *An. stephensi* to persist in artificial habitats creates opportunities for populations to survive and thrive throughout the prolonged dry season of Ethiopia. Therefore, understanding the seasonal transition between rainy and dry periods is critical to identify better approaches for *An. stephensi* control.

Larval source management (LSM) combines a suite of methods that aim to prevent the development of mosquito immature stages, such as habitat modification/manipulation (reducing the availability of larval habitats) or larviciding/biological control (adding substances or organisms that either kill or inhibit the development of larvae) [24]. Particularly for malaria prevention, LSM has shown highly variable levels of impact [25], and the World Health Organization (WHO) recommends that larviciding only be implemented in areas where larval habitats are ‘few, fixed and findable’ [24]. In India, larval control of key *An. stephensi* larval habitats such as rice paddies and artificial containers has led to important reductions in *Plasmodium* infection in mosquitoes and humans [26]. However, the challenges of implementing LSM in African cities invaded by *An. stephensi* are multiple and include largely unknown distribution of larval habitats within cities, the extent of coverage required for covering large urban centres, and limited evidence that methods that worked in the Asian context would work in Africa. An interrupted time-series analysis of malaria cases before and after a LSM intervention in Ethiopia suggested no intervention impact despite significant and persistent reductions in mosquito larval positivity and productivity [27]. The low residual activity of the larvicide formulation used (*Bacillus thuringiensis israelensis*, Bti), the presence of highly productive habitats and the potential for malaria incidence increases not being attributable to *An. stephensi* (but to an overall national increase of malaria in Ethiopia post-COVID-19) were listed as factors limiting the robustness of findings.

The persistence of *An. stephensi* in its invasive range is likely influenced by a complex interplay of factors, including the availability of suitable larval habitats, favorable climatic conditions, and the abundance of blood sources. A largely unanswered question is how populations persist in the markedly seasonal weather of Eastern Ethiopia, where drought conditions could extend for months. This study focused on the seasonal fluctuation in *An. stephensi* populations by investigating the transition in larval productivity between the rainy and dry seasons in a cohort of 100 households from Kebri Dehar, the locality in Eastern Ethiopian where *An. stephensi* was first detected in the country [4]. A secondary goal involved the study of habitat overlap between *An. stephensi* and *Aedes aegypti*, given both inhabit similar container types. To understand the implications of seasonality of *An. stephensi* populations for LSM interventions, field observations were incorporated into a mathematical model accounting for variations in larval habitat productivity and seasonality in rainfall. This model explored the potential of LSM during the dry season as a strategy for reducing or locally eliminating *An. stephensi* populations in heterogeneous environments. The study’s central hypothesis was that, in its transition into the dry season, *An. stephensi* will persist in large habitats and disappear from a wide array of small containers and surface water, forming a seasonally-driven source-sink meta-population that can make LSM interventions more efficacious.

## Methods

**Fig. 1:**
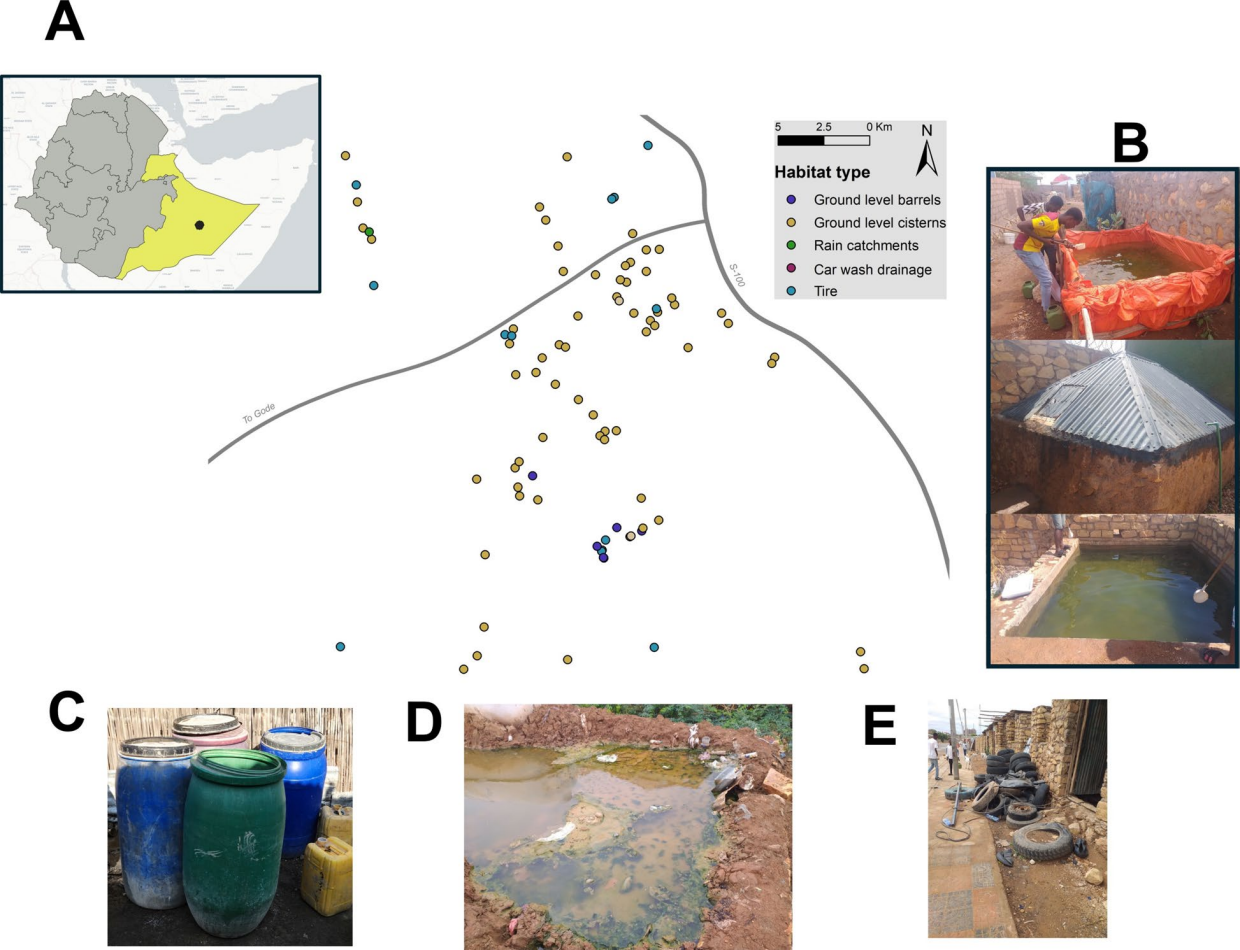
Study area and habitat characteristics. **A** Map of Kebri Dehar with the location of the 90 water-holding containers that were followed from the end of the rainy season (November 2020) and until the peak of the dry season (February 2021). Inset shows the location of Kebri Dehar within the Somali region (yellow) of Ethiopia. **B** Ground level cisterns (they could be used for construction [top panel] or residential water use with different levels of cover). **C** Plastic containers (100 or 200L drums). **D** Car wash drain areas. **E** Discarded car and truck tires. In B, images portray investigators (SY and DD) collecting larvae

**Study area.** Kebri Dehar (Fig. 1) has a population of ~ 120,000 and is distanced 400 km from Jigjiga City (Somali Region capital) and 1035 km from the capital city of Addis Ababa. The majority of the study area's geography is lowland plains, with an average elevation of 525 m above sea level and a few steeper foothills. The latitude and longitude of the area are 6° 44′ 25′′ N/44° 16′ 38′′ E, respectively. There are few shrubs and trees in the city, including several *Acacia* species and incense trees. Kebri Dehar is a semi-arid area and the average annual temperature in the area is between 23 and 30 °C. The rainfall pattern in the area is bimodal, with peaks in May and November, and averages 200 mm. Despite these patterns, the rainfall is variable, making the area prone to recurrent droughts that can last several months. Typically, each house contains an outdoor constructed cement tank used for storing water, referred to as a *birket* (Fig. 1). Other typical structures used to harbor water are also shown in Fig. 1. The house type of the study area is mainly rectangular with a corrugated iron roof and cement walls and often inhabited by multiple families.

## Study design

The initial sampling involved any potential artificial larval breeding habitat with water such as ground level cisterns, ground level barrels, plastic containers, car wash drains and rain catchments (Fig. 1). To reach the target number of 100 houses, the field team moved to the centre of Kebri Dehar and began contacting random houses asking for permission to conduct an initial survey. If a water-holding container was found, homeowners were asked to verbally consent to repeated surveys of their house for the months of December 2020, January 2021, and February 2021. Of the 100 houses visited, 15 did not have any potential habitats with water (tires, drums and cisterns were completely dry), leaving 85 houses and 90 habitats for the study. All containers with water in November 2020 were geocoded (GPS Garmin eTrex). During each survey, a habitat was first visually inspected for the presence of water. When water was present, 20 standard dips were taken (350 ml capacity) from each identified habitat by sampling its entire area. Distance to the nearest house, vegetation coverage, volume of water in the containers and habitat type (i.e., ground level cisterns, ground level barrels, plastic containers, car wash drains and rain catchments, Fig. 1) were recorded. The *Anopheles* larvae were separated from the culicine larvae based on morphology and allowed to emerge to adult in cages separated by habitat for species identification using a dissecting microscope and a standard keys [28]*.* Pupae were also separated, allowed to emerge, and identified as adult following the same methods. Kebri Dehar populations were molecularly characterized previously, and *An. stephensi* was the only Anopheline species in the habitat types sampled [4, 29, 30].

## Statistical analysis

Larval productivity was quantified as the total number of larvae present in the 20 dips per habitat and month. Heterogeneity in habitat productivity was quantified in two ways. A negative binomial fit to the distribution of larvae per container (per 20-dips) was used to estimate (via Maximum Likelihood) the parameter k, which is a measure of overdispersion in the data [31]. Values of k < 0.5 indicate strong overdispersion [31], meaning most of the larvae are aggregated (i.e. found in a few containers). The Pareto fraction [32] was used to further quantify heterogeneity. We define Pareto fraction for the number of larvae per site (*N*_*Li*_) from its empirical CDF (eCDF) as Cooper et al. [32], with *X* denoting the proportion of *N* observations and *Y* the empirical cumulative distribution: $Y\left(X\right)= {\sum }_{i<XN}{x}_{i}$. *Y(X)* monotonically increases with *X* and intersects the line 1-*X*. The point *X* where *Y(X)* = 1-*X* defines the Pareto fraction. At that point, there is a *X*:1-*X* rule, for instance if *X* = 0.8 then the Pareto fraction corresponds to the 80–20 rule. The Pareto index for a given fraction can be calculated as $\frac{\text{log}(X)}{\text{log}(\frac{X}{1-X})}.$ The Pareto fraction and Pareto index for each month were calculated. The larval sites that contributed to the identified Pareto fraction (e.g., the top 30% of sites that contribute to 70% of larvae collected) were defined as ‘superproductive’ habitats. Superproductive habitats were mapped to understand patterns of productivity across space and time. Additionally, ordinary least squares regression was used to correlate the rank of the most productive habitats (1 being most productive and least productive in ascending order) for *An. stephensi* and *Aedes aegypti*.

## Model of seasonal LSM in heterogeneous landscapes

The Kebri Dehar data was used to parameterize a multi-patch model that explicitly incorporates heterogeneity in larval productivity to explore how the seasonal transition in *An. stephensi* populations can be exploited to improve LSM. The model developed by Smith et al*.* [33], which explicitly considers the effect of larval crowding and density-dependent competition within a multi-patch framework (see Online Supplemental Text), was expanded by incorporating seasonality in rainfall and increased larval mortality due to loss of water in certain habitat types as the dry season progressed.

Specifically, the model was structured in patches, which can contain many aquatic habitats (called pools in Smith et al*.* [33]) of a given type such as construction pits, ground cement cisterns, water storage containers at home. In this model, ${L}_{i}(t)$ represents the number of larva in patch$i \in [1, 2, \cdot \cdot \cdot , n]$, where $n$ is the total number of patches, and $M$ represents the number of adult mosquitoes in the whole system of $n$ patches, modelled together as one. The model, is formally structured as:

1 $$\left\{\begin{array}{c}\frac{{dL}_{i}}{dt}=fv{p}_{i}M-\left({\alpha }_{i}+{\gamma }_{i} + {\psi }_{i}{L}_{i}^{{\delta }_{i}} \right){L}_{i}\\ \frac{dM}{dt}= {\sum }_{i}{\alpha }_{i} {L}_{i}-gM \end{array}\right.$$

where $i=\text{1,2},\dots ,n$ is the index for any patch, the mosquito larva reside in $n$ different patches, while the adult mosquito population is treated as a single unit. Other model parameters include: mosquito blood feeding rate $\left(f\right),$ the number of eggs laid by a mosquito each egg laying cycle $(v)$, the per-capita death rate of adult mosquitoes $\left(g\right)$, the fraction of eggs laid in Patch $i$ $({p}_{i})$, the maturity rate of mosquitoes in Patch $i$ $({\alpha }_{i})$, the Patch $i$ per-capita death rate not caused by overcrowding $({\gamma }_{i})$, the patch-specific increase in per-capita mortality in response to crowding ${(\psi }_{i})$, and the Patch $i$ mean crowding ${(\delta }_{i})$. The parameters contained in the model are described in Table S1.

For tractability and simplicity, a two-patch model where Patch 1 is ephemeral (containing water during the rainy season but rapidly evaporating as the dry season progresses, denoted with subscript E) and Patch 2 is stable (containing water throughout rainy and dry season, denoted with subscript S), was developed. Seasonality was incorporated in the model by linking parameter ${\gamma }_{i}$ to rainfall (described in Online Supplemental Text and Fig. S3). To incorporate the effects of LSM, larvicides were assumed to increase ${\gamma }_{E}$, or ${\gamma }_{S}$, depending on whether control was done targeting either or both patches. Intervention coverage (proportion of habitats within the patch that were treated) was also explicitly incorporated using the following equations:

$${\gamma }_{E }= {\gamma }_{E}\left(t\right)+ {\gamma }_{E,max} \times coverage$$

and

$${\gamma }_{S }= {\gamma }_{0}+ {\gamma }_{S,max} \times coverage,$$

where ${\gamma }_{E,max}=k {\gamma }_{E}\left(t\right)$ and${\gamma }_{S,max}=k {\gamma }_{0}$. The parameter $k$ represents the relative increase in larval mortality that can be achieved when the larvicide is applied with full coverage (coverage = 1) compared to the baseline mortality rates ${\gamma }_{E}\left(t\right)$ and${\gamma }_{0}$, which are the natural mortality rates of larva in the ephemeral and stable patches respectively, when no larvicide is applied (coverage = 0). Then, ${\gamma }_{. , max}$ is the maximum additional patch-larval mortality that can be induced by larvicide application, and it is proportional to the natural or baseline patch-larval mortality rate (SI Text). For this study, LSM was simulated using$k=20$. Sensitivity analyses of k were conducted to understand how larvicide efficacy impacted results (Online Supplemental Figure S5). To minimize the effects of the intervention on populations that were not yet in equilibrium, the model was run for two years, implementing LSM in the beginning of the second year.

## Results

### Characterizing the seasonal transition of *Anopheles stephensi*

A total of 90 water holding containers and potential larval habitats were identified from 100 residences surveyed from Kebri Dehar in November 2020 (Table 1). The majority (75%) of habitats were ground level cisterns (a mix of large water reservoirs used for household needs or construction purposes). During the first survey, *An. stephensi* habitat positivity (the proportion of habitats with any immature stage) was 61% (55/90 habitats) whereas *Ae. aegypti* positivity was 47% (42/90 habitats). When broken-down by habitat type, the most frequently positive habitat in the initial survey were tires (75%), followed by ground level tanks (used for residential and construction purposes) (61%), and ground level barrels (50%). A total of 3,579 immature *An. stephensi* were collected during the initial survey, followed by 822 *Ae. aegypti*, and 342 *Culex* spp. larvae.

**Table 1:** Description of each potential larval habitat type identified from Kebri Dehar (Somali region, Ethiopia) between November 2020 and February 2021

		Mean distance to house (m)			Number with water (sampled with 20 standard 350 ml dips)			
Row Labels	Count of Type		Mean area (m2)	Mean volume (L)	November	December	January	February
Ground level cisterns	68	6	11	873	70	68	70	70
Tires	12	8	6	8	12	0	0	0
Ground level barrels	6	5	7	268	6	6	6	6
Rain catchments	1	15	4	20	1	0	0	0
Car wash drainage	1	3	2	40	1	1	1	1
Total	90	7	10	683	90	75	77	77

**Fig. 2:**
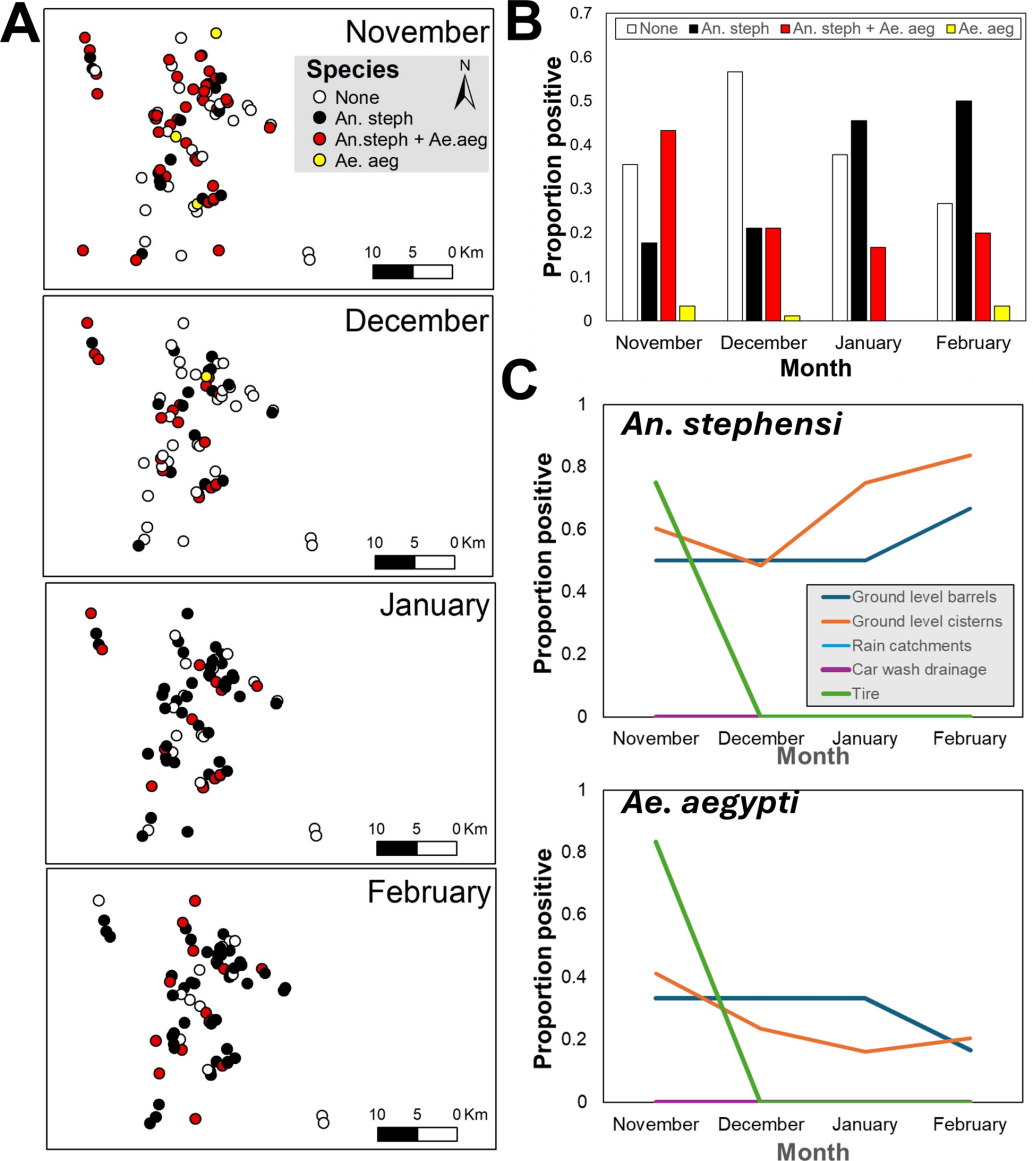
Seasonal transition in positivity of *An. stephensi* and *Ae. aegypti* in Kebri Dehar, Ethiopia. **A** Map of sites positive by each species alone or together throughout the seasonal shift. **B** Positivity (measured as proportion of habitats with either species alone or together) throughout the seasonal shift. **C** Positivity by type of larval habitat for each species

From the initial rainy season survey, and throughout the dry season, 13,635 *An. stephensi*, 1168 *Ae. aegypti*, and 757 *Culex* spp. larvae were collected. Habitat positivity varied throughout the transition of rainy to dry season (Fig. 2A). For *An. stephensi*, positivity decreased from 61% (55/90) in November to 53% (38/72) in December, followed by an increase to 74% (52/70) in January and 82% (63/77) in February (Fig. 2B). Conversely, *Ae. aegypti* positivity reduced and maintained reduction from 47% in November to 28% in December, 20% in January and 21% in February (Fig. 2B). Changes in positivity by both species were habitat- and species-specific (Fig. 2C). Whereas for *An. stephensi* the seasonal increase in positivity was primarily due to increase in the number of positive cisterns and construction pits (Fig. 2C), for *Ae. aegypti* the marked seasonal reduction was driven by a large number of tires and small larval habitats drying-up (Fig. 2C).

### Quantifying *Anopheles stephensi* habitat productivity

**Fig. 3:**
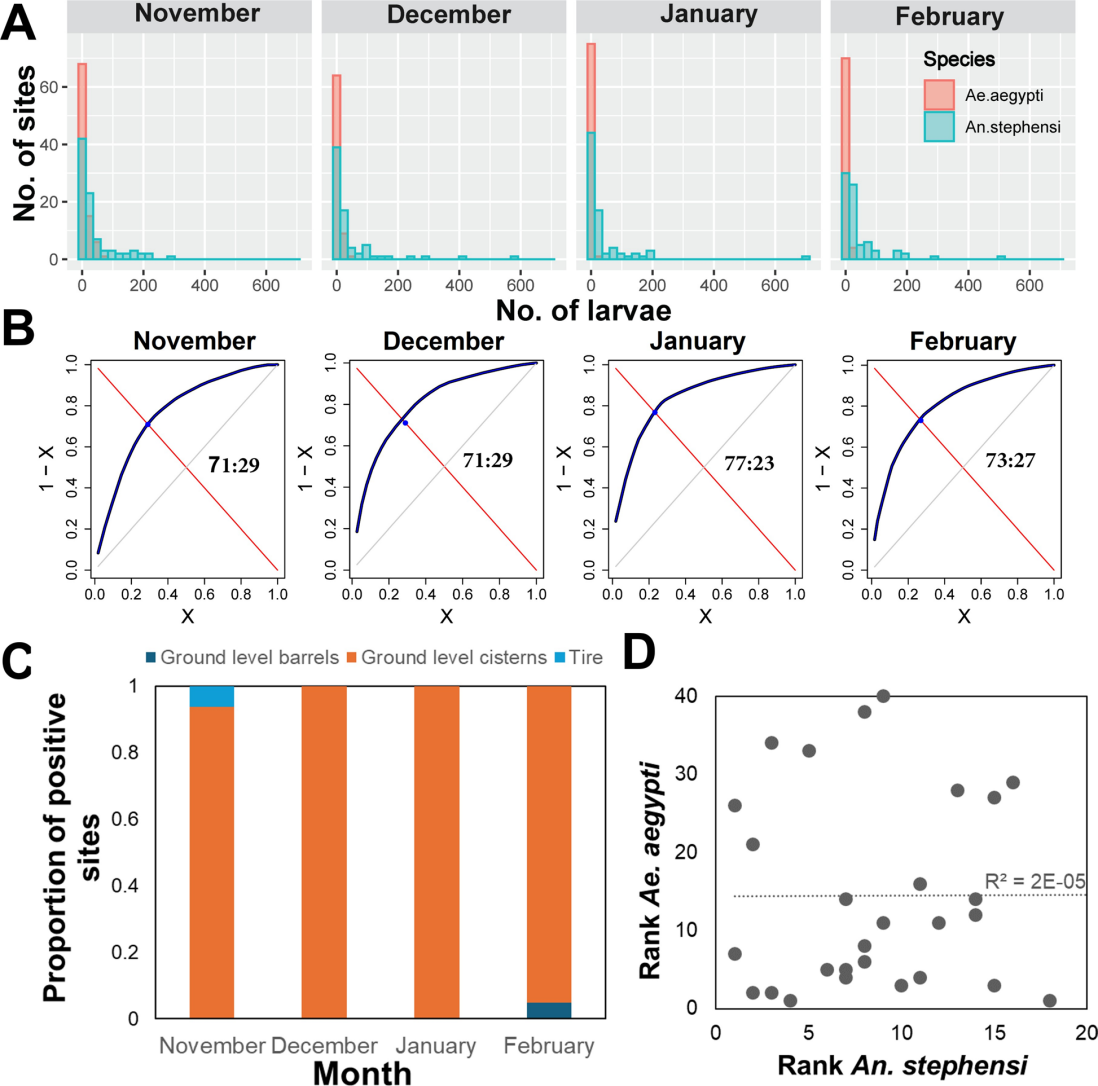
Heterogeneity in *An. stephensi* and *Ae. aegypti* larval productivity. **A** Histogram of the frequency of sites with a given number of larvae per 20 dips. **B** Results of the Pareto function applied to the number of *An. stephensi* per positive habitat. Numbers inside each panel indicate the pareto fraction (% of larvae: collected in % of larval sites). **C** For the top larval sites identified using the Pareto function, the type of habitat where *An. stephensi* was collected. **D** Rank of the top *An. stephensi* most productive larval habitats as a function of the top *Ae. aegypti* most productive habitats. The line indicates the result of a linear regression fit, with R^2^ shown inside the figure

*Anopheles stephensi* larval productivity (number of larvae per 20-dips per site) was highly heterogeneous (Fig. 3A) and was significantly predicted by a negative binomial distribution on all months (Fig. S5). When exploring the parameter *k* of the negative binomial (which measures the extent of heterogeneity in the data), evidence of extreme overdispersion (parameter *k* < 0.5) in *An. stephensi* productivity was observed on all months (Fig. S5).

The dramatic heterogeneity in larval productivity observed for *An. stephensi* was further studied by calculating the Pareto fraction (Fig. 3B). Overall, significant heterogeneity was detected across all months. In November, 29% of containers contributed 71% of *An. stephensi* larvae (71:29 in Fig. 3B). Heterogeneity increased slightly as the dry season progressed, from 71:29 in November and December to 77:23 in January and 73:27 in February (Fig. 3B). All such values indicate that most of the *An. stephensi* larvae concentrate in a few larval sites, with ground level cisterns as the primary habitat where *An. stephensi* concentrates (Fig. 3C). Comparing the ranking of superproductive sites (in decreasing order of larval productivity) between species led to the conclusion that *An. stephensi* and *Ae. aegypti* primary producing sites do not overlap (Fig. 3D). A large proportion (7/11, 64%) of *An. stephensi* superproductive sites in November were found to continue being superproducers on at least one of the subsequent months (Fig. S6). No larval site was found to be superproductive throughout all months, and only 5 (13%) sites were found to be superproductive in three out of the four months (Fig. S6).

### Implications of heterogeneous productivity for LSM

**Fig. 4:**
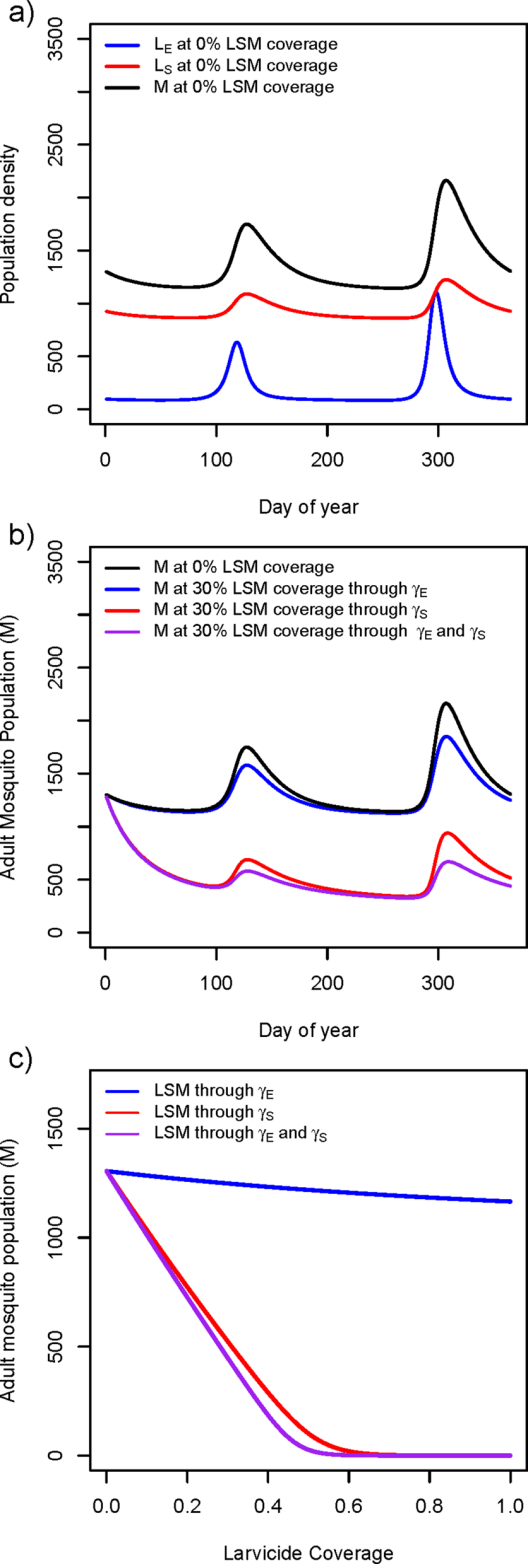
Numeric results of an *An. stephensi* population model that incorporates seasonality, two-patch dynamics, and the impact of larval control. **a** The dynamic of mosquito populations in the ephemeral (E) and stable (S) patches for both larvae (L_i_) and adult mosquitoes (M) when no control is implemented. **b** Simulating the impact of larval control at 30% coverage in the E, S and E + S patches on the number of adult mosquitoes in the entire population. **c** Impact of larval control on the adult mosquito population as a function of intervention coverage for interventions targeted at the E, S and E + S patches

The Kebri Dehar findings imply the existence of two types of habitat patches for *An. stephensi* larval persistence: stable habitats that remain with water and produce large numbers of larvae throughout the year (e.g., cisterns and construction pits) and ephemeral habitats that either dry-up or lower their productivity as the dry season progresses (e.g., tires, small buckets, ground-level barrels). Modelling *An. stephensi* population dynamics using default parameters (Table S1 and Kebri Dehar rainfall) in both habitat patches reproduced the similar magnitude difference in larval productivity observed between them in the field as well as clear seasonality in adult abundance driven by rainfall (Fig. 4A).

Simulating the implementation of residual larviciding during the dry season (February) with a larvicide that lasts up to six months and at a coverage of 30% led to a minor reduction in *An. stephensi* adult density when the ephemeral patch was treated (increase in γ_E,_ Fig. 4B). When the stable patch was treated at the same coverage and with the same larvicide, a dramatic reduction in adult mosquito population was observed (increase in γ_S_, Fig. 4B). Interestingly, simultaneously applying LSM on both the stable and ephemeral patches (increase γ_S_ and γ_E_) led to a very similar reduction in adult mosquitoes than controlling the stable patch alone (Fig. 4B). Each intervention strategy showed a different impact on the adult population as the intervention coverage increased (Fig. 4C).

Controlling the ephemeral patch led to minor impact on adult mosquitoes (~ 10% reduction). When the intervention was conducted on the stable patch, adult mosquito population size was exponentially reduced, with vector elimination occurring at LSM coverages above 60% (Fig. 4C). Interestingly, controlling both ephemeral and stable patches led to vector elimination at 50% coverage of all habitats, a minor vector control gain compared to targeting the stable patch alone (Fig. 4C).

## Discussion

During the rainy season, *An. stephensi* is commonly found in a wide array of larval sites throughout Ethiopia [22]. Conversely, during the peak of the dry season in Jigjiga (Somali Region) *An. stephensi* was found infesting a few locations, primarily ground level cisterns associated with construction [20]. Unifying such findings, this study shows that *An. stephensi* undergoes a major population transition between the rainy and dry seasons, concentrating in ground level cisterns and disappearing from smaller artificial containers due to them drying out. A similar pattern of persistence of *An. stephensi* during the dry season was reported from Kenya, where vector densities were much smaller (total of 114 larvae from a multi-district collection effort) than in this study [34]. While positivity was informative for identifying the array of potential larval habitats, larval productivity became more relevant for identifying the potential contribution of each site to the maintenance of *An. stephensi* populations.

Significant levels of heterogeneity in *An. stephensi* larval productivity, evidenced by the contribution of up to 77% of all larvae by only 23% of the larval sites, were quantified. The trend of aggregation was consistent across months, with an observed slight increase in the level of heterogeneity as Kebri Dehar transitioned from the rainy to the dry season. Using a Pareto function, the fraction of habitats that contributed the most larvae was identified and referred as “superproductive” to separate them from negative or low-density habitats. In the context of this study, a superproductive habitat contributes to the majority of immature *An. stephensi* mosquitoes within a survey month and is identified by ranking the number of larvae per habitat from highest to lowest and selecting sites until the Pareto fraction value (e.g., 77%) was reached. A simpler method could involve selecting the habitats until a benchmark value of 70% is reached. More complex approaches, which may involve comparing the k of the negative binomial to the expectation of a Poisson distribution may be conducted, as done for infectious disease superspreading [35]. Lindblade et al*.* [36] implementation of a sequential sampling based on the Wald’s probability ratio test would allow implementing larval surveys to identify whether a habitat is indeed superproductive (upper limit of selection), low density (lower limit of selection) or negative. Unfortunately, *An. stephensi* adult collection numbers in Ethiopia tend to be very low and centred in peridomestic habitats [22, 29], limiting our ability to correlate superproductivity with adult mosquito density. While a correlation between heterogeneity in *An. stephensi* adult density and malaria transmission potential are likely (as observed for *An. gambiae* in Uganda [32]), future studies should focus on superproductive habitats and their potential role as sources for adult *An. stephensi* and increased risk of urban malaria transmission.

In Africa, *Ae. aegypti* has been reported anecdotally cohabiting with *An. stephensi* in various larval sites [19, 22, 37]. This study shows that the level of overlap varies with the season and the type of habitat. During the rainy season, both species exploit similar containers (plastic containers and tires) and can be found in other habitats not surveyed in Kebri Dehar [23]. As the dry season progresses, the lack of rain coupled with high heat leads to rapid water evaporation in small containers and tires and to the disappearance of both mosquito species from them. It is possible that *Ae. aegypti* survives the long dry season due to the large crop of eggs remaining in dried-out plastic containers and tires. As water holding containers remaining in the dry season are primarily ground cisterns and large plastic drums, it is possible that *Ae. aegypti* faces additional sources of mortality due to other mosquito larvae, predators and increased sun irradiation in open cisterns and construction pits. Combined, such factors may be responsible for the low productivity of *Ae. aegypti* and the high productivity of habitats by *An. stephensi*. More research on the larval ecology of both species is needed to understand whether cohabiting can be used to leverage LSM implementation. Findings from this study suggest that the most productive habitats for *An. stephensi* are not necessarily the most productive habitats for *Ae. aegypti*, challenging the idea of efficient control of both species if specific habitats are targeted.

Several limitations impacted the quality of this study. First, sampling focused on 100 houses with 90 larval habitats and it is likely specific larval sites that have been described by others as infested by *An. stephensi* during the rainy season (e.g., buckets and small plastic containers) were missed. Given what was described for tires and other containers, those small habitats are very likely prone to drying and may not contribute to significant productivity in the dry season. Second, pupae are often used to quantify productivity. Unfortunately, this study only detected pupae in a few instances (80 sites out of 319 with water) and the field team was unable to identify all of them to species. Therefore, more complex measures of productivity (pupae per dip, pupae per person [38]) were not attempted and should be the focus of future research.

Considering that *An. stephensi* eggs only survive desiccation for a few days [39] the finding of increased heterogeneity in the dry season could be of significant relevance for LSM deployment. The empirical and modelling findings imply that *An. stephensi* populations may be shaped by seasonally-driven metapopulation dynamics. While in the rainy season population mixing is high and occurs across both stable (e.g., large cisterns, construction pits and other large water reservoirs) and ephemeral (e.g., tires, small containers and other water reservoirs) habitats, in the dry season stable habitats which are replenished by filling tap water from time to time function as sources of adult mosquitoes that maintain the population throughout the dry season. Given this strong seasonality in productivity, the model suggest that targeting larval control in stable habitats during the dry season can lead to significant population reductions and even vector elimination if coverage is above 60%. The model also shows how sensitive findings may be to the efficacy of the larvicide. As larvicides such as pyriproxyfen [40] and spinosad [41], which have long-lasting formulations that last up to six months, continue being evaluated and showing high efficacy against *An. stephensi*, the opportunity for sustained long-term control becomes more attainable. Other forms of control, including mosquito proofing water cisterns [42] or the introduction of larvivorous fish [43] may be biorational alternatives to the use of larvicides, particularly in stable habitats that remain with water for years. The findings from this study contradict an early interrupted time series analysis of an LSM campaign against *An. stephensi* that found no change in malaria incidence due to the intervention [27]. Such study did not include any measure of heterogeneity in larval distribution or habitat productivity, relied on the analysis of an intervention with a larvicide of poor duration (Bti), and did not link malaria cases to their location of residence or the presence of *An. stephensi*, which may be the main reason why control would have been ineffective. The findings from the model, if validated empirically with field trials, suggest that the goal of *An. stephensi* elimination from Ethiopian cities with precision LSM at adequate coverage may be within reach.

Some challenges potentially faced in the deployment of LSM in Ethiopia is the limited infrastructure for urban vector control and the community acceptability of the intervention. Pilot evaluations of LSM using biological larvicides (*Bacillus thuringiensis*, Bti) by US President’s Malaria Initiative (PMI) led to important entomological impacts but also evidenced the immense infrastructure needed to map, identify and treat every single habitat with a larvicide lasting two weeks [44]. Conducting monthly applications of BTI (VectoBac WG) led to a reduction of *An. stephensi* from 18.7 larvae per 20 dips at baseline to 3.3 larvae per 20 dips during the intervention [44]. Kebri Dehar was one of the localities where PMI conducted its LSM pilot implementation [44]. A key challenge of the LSM implementation was intervention sustainability; the large number of habitats and vertical nature of the intervention led to rapid attrition of field personnel and supervisors, and a sense of difficulty in maintaining the intervention should it be transferred to the local governments [44]. As observed for *Ae. aegypti* and dengue control, communities can play an important role in supporting source reduction and other LSM approaches against *An. stephensi* [45]. Approaches embraced by WHO such as Communication for Behavioural Impact (COMBI) [46] may provide important framework for the control of *An. stephensi*. Educating urban communities about the threat of *An. stephensi* (and *Ae. aegypti*) and what steps can be taken to reduce their presence (covering open containers, avoiding the accumulation of water during the rainy season, treating water used for human consumption) could expand LSM from simple find-and-treat to a more integrated intervention in which communities are included and engaged.

## Conclusions

*Anopheles stephensi* invasion of Africa has led to significant challenges for local and national malaria control programmes aiming at containing this urban container-breeding mosquito. This study provides important information about *An. stephensi* larval ecology and the potential for vector elimination through data-informed and targeted LSM. In the highly seasonal urban centres of Ethiopia, *An. stephensi* experiences a major population bottleneck during the dry season, concentrating productivity in large water cisterns associated with residential water consumption or construction. The stability of nutrient-rich water throughout the year makes cisterns and construction pits ideal productive habitats, leading some of them to become superproductive (they contribute to the majority of immature *An. stephensi* larvae across all infested sites. A two-patch metapopulation model suggested that targeting LSM with a long-lasting larvicide on such stable habitats at a coverage above 60% could lead to major population reductions and vector elimination even if ephemerous habitats are left untreated. Such finding can have profound implications for the design of LSM plans against *An. stephensi* in Ethiopia and beyond.

## Supplementary Information

Additional file1

Additional file2

## Data Availability

All data have been made available as a supplementary file to this manuscript. The file Database.xlsx contains all the information to recreate the results in this paper.

## Abbreviations

Bti: Bacillus thuringiensis israelensis
CDF: cummulative density function
COMBI: Community Mobilization for Behavioural Impact
GPS: Global Positioning System
LSM: Larval Source Management
PMI: President’s Malaria Initiative
WG: Wettable granules
WHO: World Health Organization
